# Comment on “Three New Dimers and Two Monomers of Phenolic Amides from the Fruits of *Lycium barbarum* and Their Antioxidant Activities”

**DOI:** 10.1021/acs.jafc.3c08738

**Published:** 2024-03-12

**Authors:** Annemiek van Zadelhoff, Wouter J.C. de Bruijn, Jean-Paul Vincken

**Affiliations:** Laboratory of Food Chemistry, Wageningen University & Research, Bornse Weilanden 9, 6708 WG Wageningen, The Netherlands

## Abstract

This Comment critically addresses the article by Gao *et al*. (GaoK., *et al*. J. Agric. Food Chem.2015, 63, 1067−107525603493 10.1021/jf5049222), providing the structural elucidation of three phenolamide dimers (neolignanamides) from the fruits of *Lycium barbarum*. A more recent article published by Chen *et al*. (ChenH., *et al*. J. Agric. Food Chem.2023, 71, 11080−1109337462007 10.1021/acs.jafc.3c01669) incorporates these structures into further research on the bioactivity of these compounds. Although the analytical techniques used by Gao *et al*. are adequate, in our opinion, the nuclear magnetic resonance (NMR) spectroscopic data have not been interpreted correctly, resulting in incorrect structures for three neolignanamides from the fruits of *L. barbarum*. In this Comment, an alternative interpretation of the NMR spectroscopic data and the corresponding structures are proposed. The proposed structures feature linkage types that are much more common for neolignanamides than the linkage types in the originally reported structures of these compounds.

Neolignanamides are dimers of phenolamides, monomers consisting of a wide variety of phenylalkenoic acid (usually a hydroxycinnamic acid) and amine subunits that can be linked by at least 14 known linkage types, resulting in a large number of possible neolignanamide structures. For all neolignanamides reported thus far, the coupling occurs via the hydroxycinnamic acid moiety of the phenolamide.^1^ Because these compounds are known to possess various bioactivities, many articles have described the purification of these compounds from plants, identified by nuclear magnetic resonance (NMR) spectroscopy, and tests of their bioactivity. Gao *et al*.^2^ reported three newly identified feruloyltyramine (FerTrm) dimers [compounds **3**–**5** (Figure 1)] from the fruits of *Lycium barbarum*. For FerTrm dimers, at least eight types of linkages between the monomers have been reported, all of which solely involve the hydroxycinnamoyl moiety.^1^ The structures published by Gao *etal*. feature a highly unusual linkage via the amine moiety. To the best of our knowledge, this is the only report of phenolamide coupling via the amine moiety. The general consensus regarding the mechanisms underlying formation of neolignanamides is that coupling occurs after initial radical formation on the *p*-hydroxyl group of the phenolamides’ hydroxycinnamoyl moiety.

**Figure 1:**
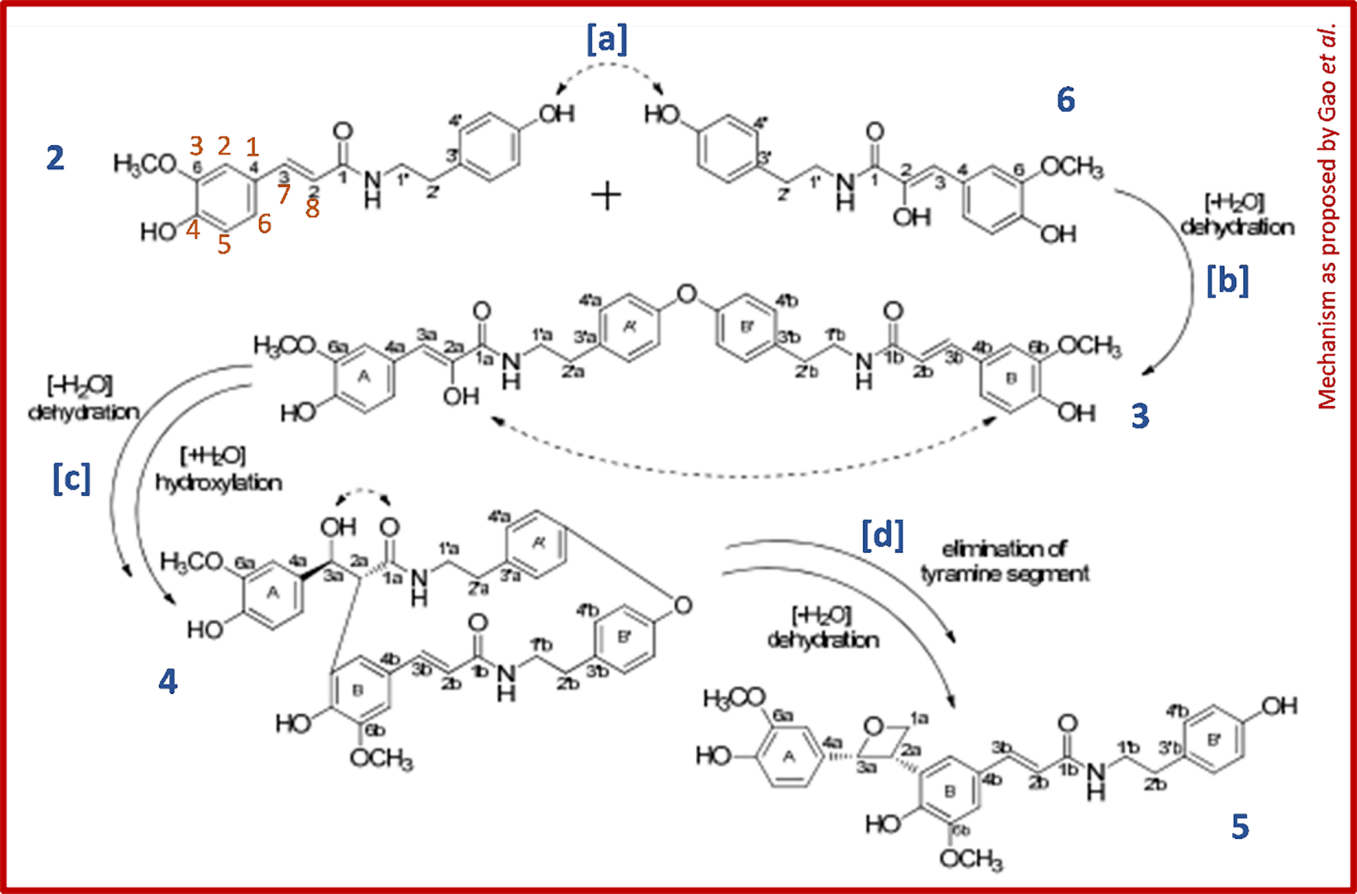
Putative mechanism as published in the Supporting Information of Gao *et al*.,^2^ adapted with added (blue) compound numbers and letters indicating the different reaction steps and (orange) generally accepted atom numbering that will be used throughout this Comment.

The article provides a putative biosynthetic route for the formation of these linkage types (Figure 1), which lacks detailed information and a chemical rationale for the underlying reaction mechanisms. The reported dimers were proposed to be coupling products of FerTrm (**2**) and 8-hydroxy-FerTrm [(8_OH_)-FerTrm (**6**)]. However, (8_OH_)-FerTrm has not been reported elsewhere and was not identified in the fruits of *L. barbarum* by the authors either. These two precursors were suggested to couple by the two *p*-hydroxyl groups of the tyramine moiety attacking each other (step [a]), which seems highly unlikely. Moreover, instead of the formation of an −*O*–*O*– bond, an ether linkage is formed (step [b]), resulting in the suggested structure of compound **3**. Compound **4** was suggested to be formed via intramolecular attack of the *C*8-hydroxyl group of the hydroxycinnamoyl moiety on the benzene ring of the other hydroxycinnamoyl moiety (step [c]), oddly resulting in the formation of a carbon–carbon bond and the spontaneous migration of the *C*8-hydroxyl group of the hydroxycinnamoyl moiety to the neighboring *C*7. The final step of the mechanism (step [d]) involves multiple unexpected reactions. First, the ether bond connecting the two tyramine moieties is broken without a clear driving force. Second, the amine bond of one of the tyramine moieties is eliminated spontaneously. Third, intramolecular attack of a hydroxyl group on a carbonyl releases one of the oxygen atoms, resulting in the formation of a four-membered heterocycle. The proposed mechanism lacks an explanation of the driving forces and electron movements underlying these reactions. The unusual linkage types and highly unlikely mechanism proposed by Gao *et al*. prompted a more in-depth investigation of their NMR spectroscopic data.

Upon checking the NMR spectroscopic data of the compounds, we found that the structure elucidation was incorrect. The NMR spectroscopic data provided for compound **3** match the NMR spectroscopic data for the previously reported compound cannabisin F^3^ and a dimer from *Peperomia tetraphylla*(4) (Table 1), both of which are FerTrm dimers formed via a 4-*O*-8′-linkage.^3,4^ The NMR data of Gao *et al*.^2^ were obtained in CD_3_OD, whereas acetone-*d*_6_ was used in other studies reporting NMR data for FerTrm dimers formed via a 4-*O*-8′-linkage. The same issue occurred for compounds **4** and **5** (Figure 2). This could affect the NMR shifts to some extent; however, the difference between these two solvents is expected to be limited.^5^ Because the two-dimensional (2D) NMR data provided by Gao *et al*.^2^ were used to determine the structures of the compounds, the difference in the solvent used does not affect the identification of these compounds. The comparison to other literature on compounds with the same 2D structure is used only as an additional confirmation of the structure. The key correlations that would be expected for this linkage type were also detected in the HMBC spectrum of compound **3** (Figure 3). Additionally, the 2D NMR spectroscopic data contained correlations that are typical for the 4-*O*-8′-linkage and that are also reported for other neolignanamides with the same linkage type, e.g., 4-*O*-8′-linked feruloylagmatine,^6^ corydalisin A and B,^7^ and melongenamide C.^8^ Furthermore, all of the proton signals that would be expected for an unsubstituted FerTrm unit^2,9^ were detected. This indicates that none of its carbon atoms are involved in the linkage for one of the FerTrm moieties, as this would have resulted in the loss of at least one proton signal. In addition, the absence of the *H*8′ proton signal, and the *H*7′ proton signal being a singlet, also confirm the 4-*O*-8′-linkage. The NMR chemical shifts corresponding to the hydroxycinnamoyl moieties of these dimers also matched the chemical shifts corresponding to the hydroxycinnamoyl moieties reported for a 4-*O*-8′-linked feruloylagmatine dimer.^6^ These compounds differ in the amine moiety (i.e., tyramine vs agmatine); thus, a match in the NMR spectroscopic data of the hydroxycinnamoyl moieties further supports the idea that the linkage is formed via the hydroxycinnamic acid moiety rather than the amine moiety.

**Figure 2:**
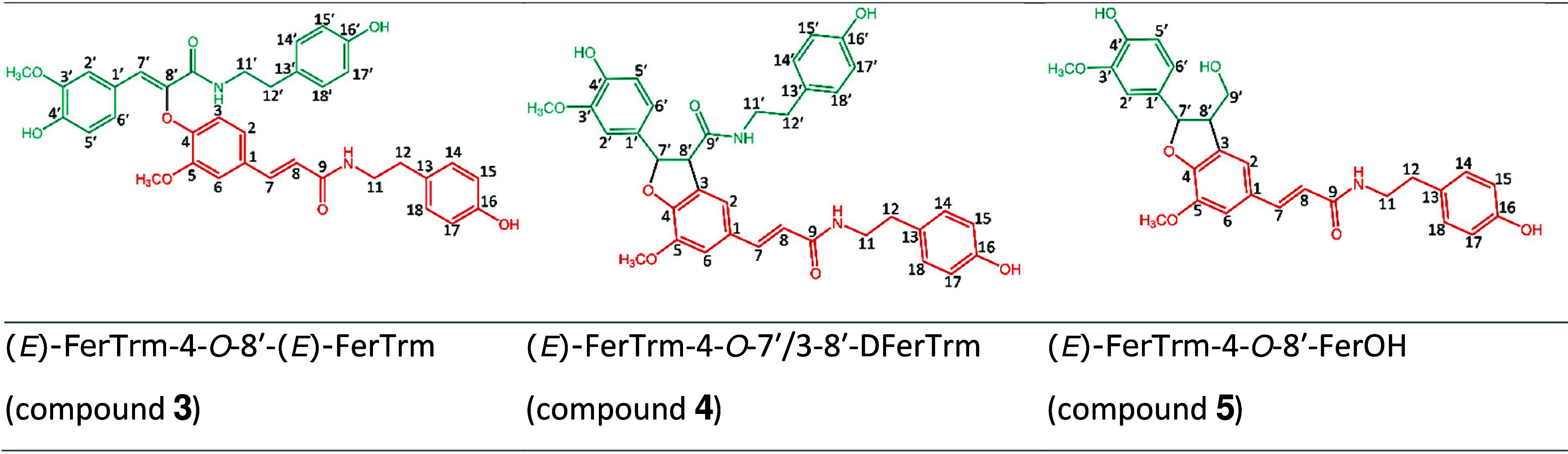
Suggested structures of compounds **3**–**5**.

**Table 1:** Comparison of ^1^H and ^13^C NMR Spectral Data for Compounds **3–5** with Those of Previously Published (Neo)lignanamides

	compound **3**^2^		cannabisin F^3^		compound **4**^2^		grossamide^11^		compound **5**^2^		grossamide K^11^	
	^1^H (500 MHz) and ^13^C NMR (125 MHz) in CD_3_OD		^1^H (500 MHz) and ^13^C NMR (125 MHz) in acetone-*d*_6_		^1^H (500 MHz) and ^13^C NMR (125 MHz) in CD_3_OD		^1^H (300 MHz) and ^13^C NMR (75 MHz) in acetone-*d*_6_		^1^H (500 MHz) and ^13^C NMR (125 MHz) in CD_3_OD		^1^H (300 MHz) and ^13^C NMR (75 MHz) in acetone-*d*_6_	
atom number	δ_C_	δ_H_ (mult., *J*)	δ_C_	δ_H_ (mult., *J*)	δ_C_	δ_H_ (mult., *J*)	δ_C_	δ_H_ (mult., *J*)	δ_C_	δ_H_ (mult., *J*)	δ_C_	δ_H_ (mult., *J*)
1	129.4		131.7		130.6		129.4		130.3		129.7	
2	112.3	7.30 (d, 1.2)	113.6	7.35 (d, 2.0)	118.3	6.77 (s)	119.0	6.44 (s)	118.8	7.16 (s)	117.8	7.14 (s)
3	149.1		150.2		129.6		129.0		131.4		130.8	
4	148.2		148.8		151.4		150.4		151.5		150.7	
5	113.7	6.72 (d, 8.0)	115.9	6.78 (d, 8.4)	146.2		145.4		145.9		145.2	
6	124.9	7.05 (dd, 8.0, 1.2)	125.5	7.14 (dd, 8.4, 2.0)	113.4	7.13 (d, 1.2)	111.0	7.07 (s)	113.7	7.10 (d, 1.8)	112.8	7.07 (s)
7	139.8	7.49 (d, 15.7)	139.6	7.46 (d, 15.7)	142.0	7.46 (d, 15.7)	141.2	7.41 (d, 15.7)	142.1	7.50 (d, 15.7)	140.7	7.49 (d, 15.6)
8	119.6	6.53 (d, 15.7)	122.0	6.57 (d, 15.7)	119.6	6.42 (d, 15.7)	118.8	6.46 (d, 15.8)	119.5	6.47 (d, 15.7)	119.8	6.54 (d, 15.6)
9	167.4		163.3		169.2		167.4		169.1		166.7	
11	40.9	3.48 (br, t, 7.0)	41.8	3.46 (dd, 13.0, 6.0)	42.7	3.50 (t, 7.2)	42.0	3.75 (m)	42.7	3.48 (t, 7.3)	42.0	3.56–3.50 (m)
12	34.0	2.65 (t, 6.7)	35.5	2.65 (t, 7.1)	35.9	2.79 (t, 7.2)	35.4	2.77 (t, 7.1)	36.0	2.77 (t, 7.3)	35.6	2.75 (t, 7.0)
13	129.9		130.8		131.4		130.7		131.0		130.9	
14	129.3	6.85 (d, 8.4)	130.5	6.90 (d, 8.6)	130.9	7.08 (d, 8.4)	130.3	7.05 (d, 8.3)	130.9	7.07 (d, 8.4)	130.5	7.06 (d, 8.7)
15	115.0	6.61 (d, 8.4)	116.1	6.68 (d, 8.6)	116.4	6.74 (d, 8.4)	115.9	6.74 (d, 8.3)	116.4	6.74 (d, 8.4)	116.0	6.76 (d, 8.7)
16	155.4		156.7		157.0		156.7		157.0		156.7	
17	115.0	6.61 (d, 8.4)	116.1	6.68 (d, 8.6)	116.4	6.74 (d, 8.4)	115.9	6.74 (d, 8.3)	116.4	6.74 (d, 8.4)	116.0	6.76 (d, 8.7)
18	129.3	6.85 (d, 8.4)	130.5	6.90 (d, 8.6)	130.9	7.08 (d, 8.4)	130.3	7.05 (d, 8.3)	130.9	7.07 (d, 8.4)	130.5	7.06 (d, 8.7)
*C*3-OCH_3_	54.5	3.67 (s)	56.0	3.69 (s)	56.8	3.91 (s)	56.2	3.83 (s)	57.0	3.91 (s)	56.3	3.85 (s)
1′	124.0		125.5		131.8		132.3		134.4		133.8	
2′	111.1	7.24 (d, 2.0)	112.3	7.27 (d, 2.0)	110.8	6.93 (d, 1.8)	110.4	6.96 (s)	110.9	6.97 (d, 1.8)	110.5	7.00 (d, 2.0)
3′	147.5		148.3		149.4		148.3		149.3		148.4	
4′	146.1		147.1		148.3		147.5		147.9		147.4	
5′	115.0	6.73 (d, 8.0)	115.1	6.75 (d, 8.4)	116.5	6.81 (d, 8.2)	115.7	6.81 (s)	116.4	6.80 (d, 8.2)	115.7	6.82 (d, 8.2)
6′	120.9	7.02 (dd, 8.0, 2.0)	121.5	7.03 (dd, 8.4, 2.0)	120.2	6.79 (d, 1.8)	119.7	6.70–6.81 (m)	120.2	6.84 (d, 1.8)	119.6	7.06 (d, 8.7)
7′	124.0	7.26 (s)	123.7	7.27 (s)	90.1	5.90 (d, 8.3)	88.8	6.02 (d, 8.6)	90.0	5.58 (d, 6.4)	88.9	5.60 (d, 6.4)
8′	140.1		142.3		58.8	4.16 (d, 8.13)	57.6	4.18 (d, 8.6)	55.1	3.54 (q, 6.2)	54.3	3.57 (q, 6.4)
9′	164.0		166.1		173.0		170.3		65.0	3.84 (m)	64.3	3.79–3.91 (m)
11′	41.2	3.48 (br, t, 7.0)	42.0	3.49 (dd, 13.0, 6.0)	42.4	3.50 (t, 7.2)	41.4	3.5–3.57 (m)				
12′	34.5	2.77 (t, 7.2)	35.7	2.75 (t, 7.1)	35.5	2.76 (t, 7.2)	34.5	2.83 (t, 6.0)				
13′	130.5		131.1		131.2		130.4					
14′	129.3	7,07 (d, 8.4)	130.5	7.06 (d, 8.6)	131.0	7.03 (d, 8.4)	130.5	6.77 (d, 6.9)				
15′	115.0	6.73 (d, 8.0)	116.1	6.75 (d, 8.6)	116.6	6.84 (d, 8.4)	116.7	6.80 (d, 6.9)				
16′	155.6		156.7		157.1		156.5					
17′	115.0	6.73 (d, 8.0)	116.1	6.75 (d, 8.6)	116.6	6.84 (d, 8.4)	116.7	6.80 (d, 6.9)				
18′	129.3	7.07 (d, 8.4)	130.5	7.06 (d, 8.6)	131.0	7.03 (d, 8.4)	130.5	6.77 (d, 6.9)				
*C*3′-OCH_3_′	55.0	3.92 (s)	56.2	3.94 (s)	56.5	3.84 (s)	56.2	3.80 (s)	56.6	3.83 (s)	56.2	3.80 (s)

**Figure 3:**
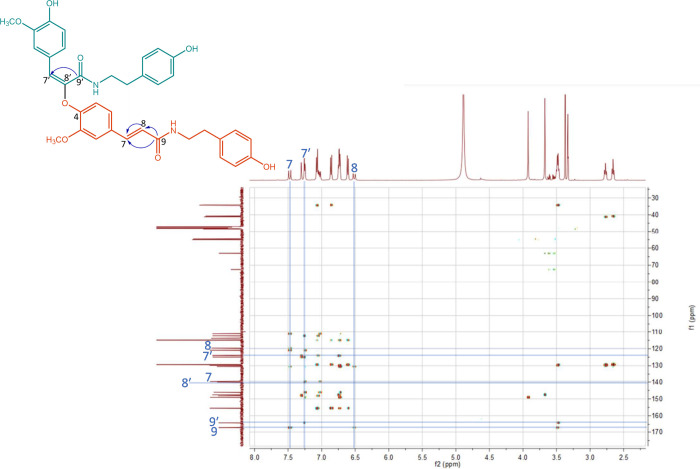
Structure of compound **3** with key HMBC correlations, and the corresponding HMBC spectrum as published in the Supporting Information of Gao *et al*.,^2^ adapted with addition of key correlations (blue lines) and atom numbers in the proton and carbon spectra (blue numbers).

We also compared the NMR spectroscopic data of compound **4** to those reported previously in the literature. This led to the conclusion that this compound is actually linked via a 4-*O*-7′/3-8′-linkage. This is a linkage type very commonly reported for (neo)lignanamides. The dimer of feruloyltyramine linked via this linkage type, known as grossamide, was previously reported with NMR spectroscopic data matching those provided by Gao *et al*. (Table 1).^10,11^ For this compound, the expected key correlations corresponding to the 4-*O*-7′/3-8′-linkage were detected in its HMBC spectrum, as well, as shown in Figure 4. Similar to compound **3**, the overlap in NMR shifts between compound **4** (a feruloyltyramine dimer) and a feruloylagmatine dimer^6^ with the 4-*O*-7′/3-8′-linkage indicated that the linkage was indeed formed at the hydroxycinnamic acid moiety and that the amine moiety is not involved. Very typical for this linkage type are the protons with δ 4.16 (*H*8′) and δ 5.90 (*H*7′) showing six and five HMBC correlations, respectively, due to their proximity to the linkage.

**Figure 4:**
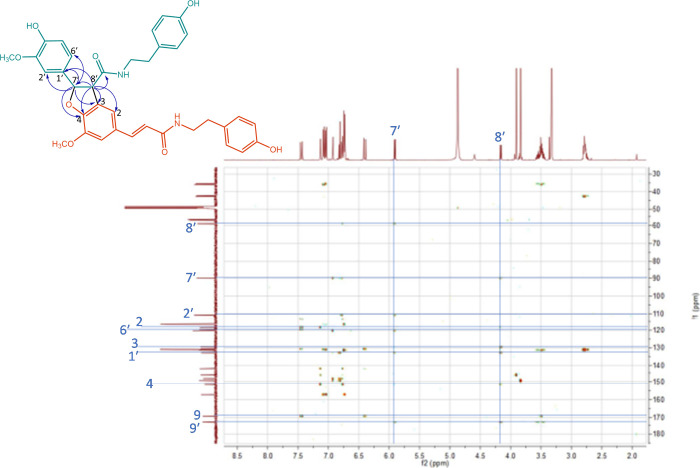
Structure of compound **4** with key HMBC correlations, and the corresponding HMBC spectrum as published in the Supporting Information of Gao *et al*.,^2^ adapted with addition of key correlations (blue lines) and atom numbers in the proton and carbon spectra (blue numbers).

Compound **5** indeed corresponds to a feruloyltyramine linked to a coniferyl alcohol as proposed by Gao *et al*.; however, on the basis of the NMR spectroscopic data, the linkage type is actually also a 4-*O*-7′/3-8′-linkage. A dimer with the same composition and linkage type has been previously reported as grossamide K (Table 1).^11^ For this compound, the key correlations expected for the 4-*O*-7′/3-8′-linkage were also detected in the HMBC data (Figure 5).

**Figure 5:**
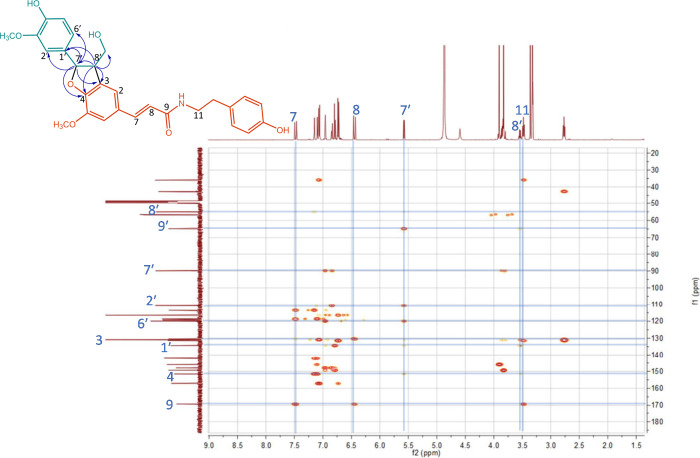
Structure of compound **5** with key HMBC correlations, and the corresponding HMBC spectrum as published in the Supporting Information of Gao *et al*.,^2^ adapted with addition of key correlations (blue lines) and atom numbers in the proton and carbon spectra (blue numbers).

Thus, on the basis of a comparison of the NMR spectroscopic data reported by Gao *et al*. to those reported elsewhere in the literature, we believe that compounds **3**–**5** should be (*E*)-FerTrm-4-*O*-8′-(*E*)-FerTrm, (*E*)-FerTrm-4-*O*-7′/3-8′-DFerTrm, and (*E*)-FerTrm-4-*O*-7′/3-8′-DFerOH, respectively (Figure 2).

These structures feature linkage types that are much more common for (neo)lignanamides than the linkage types reported by Gao *et al*. It is worth noting that compounds with these 2D structures were already previously reported in a wide variety of plants,^11−13^ including *L. barbarum*, and are also known as cannabisin F, grossamide, and grossamide K.^14^ It should be noted that we were unable to confirm, on the basis of the data reported by Gao *et al*., whether the configurations of the chiral carbon atoms in compounds **4** and **5** match the configurations of these previously reported dimers. Therefore, compounds **4** and **5** could be either identical to or stereoisomers of grossamide and grossamide K, respectively. Moreover, these structures can be formed via chemically sound and generally accepted mechanisms, which are much more plausible than the biosynthetic route suggested by Gao *et al*.

We hope to have provided sufficient information to support why we believe that the structures proposed in Gao *et al*. were incorrect and why the structures shown in Figure 2 are the actual structures of compounds **3**–**5**. Our primary motivation in addressing this issue was to avoid confusion in the literature and further continuation of research based on incorrect structures. Fortunately, the number of articles incorporating the incorrectly elucidated structures is still limited. However, one article recently published in the *Journal of Agricultural and Food Chemistry* uses the incorrect structures for a docking study,^15^ which exemplifies why it is important to rectify the elucidation of these structures.
